# Informal STEM learning: Examples from everyday spatial behaviors

**DOI:** 10.3389/fpsyg.2023.1117771

**Published:** 2023-03-10

**Authors:** Yingying Yang, Sonia Conde Santiago, Daria Lasc, Arielle Hershkovich, Lauren Grove

**Affiliations:** Department of Psychology, Montclair State University, Montclair, NJ, United States

**Keywords:** informal STEM learning, everyday spatial behavior, children, sense of direction, adaptive living, sex difference, age difference

## Abstract

**Introduction:**

Extensive research has shown a close relationship between spatial abilities and success in STEM disciplines because many STEM problems often require students to reason about spatial information. Everyday spatial behaviors may predate and facilitate the development of spatial skills. Therefore, the current study examined children’s everyday spatial behaviors and their associations with broader child development outcomes and individual differences.

**Methods:**

Based on previous research, we developed an everyday spatial behaviors questionnaire for children (ESBQC). A total of 174 parents and their children aged 4–9 years old participated. In ESBQC, parents rated how much difficulty their children experience with different spatial behaviors, such as putting together a puzzle, retracing a route, or hitting a moving ball.

**Results:**

Factor analysis revealed 8 components in ESBQC. The internal reliabilities were relatively high. ESBQC was positively correlated with age but not with sex. Furthermore, ESBQC predicted sense of direction, even after considering age and bias associated with parent reports.

**Discussion:**

Our questionnaire may provide a useful tool for parents and other stakeholders to better understand everyday spatial behaviors and encourage interest and competence in spatial skills, ultimately promoting STEM learning in informal, everyday settings.

## Introduction

The United States is facing an ever-increasing demand for talent in STEM fields (science, technology, engineering, and mathematics, U.S. Bureau of Labor Statistics, 2022). However, fewer students than needed are pursuing certain STEM majors and careers (Xue and Larson, 2015). Furthermore, many K-12 students (as high as 30%–40%) are not meeting national STEM content standards (U.S. Department of Education, 2019). Therefore, there is an urgent need to increase STEM competency and the number of people going into STEM fields. STEM interests and identities can and should be fostered early in child development (Perez et al., 2014; Dou et al., 2019), with STEM learning occurring in formal school settings and informal settings outside of school (Alexandre et al., 2022). Researching informal STEM learning is particularly critical, considering that children spend 80% of their time outside school (Meltzoff et al., 2009). The current study examined 4–9-year-olds’ informal STEM-related activities through parent reports of everyday spatial behaviors.

### Spatial abilities and everyday spatial behaviors

Spatial abilities refer to representing, manipulating, and remembering the visual–spatial relations among objects or space. People perform spatial behaviors every day, which commonly draw on spatial abilities. For instance, a common spatial behavior, such as putting together a puzzle, may involve the spatial abilities of spatial perception and mental rotation. Everyday spatial behaviors can be examined *via* questionnaires. For instance, Newcombe et al. (1983) asked college students to rate how often (1: never, 6: more than once a week) they participated in several spatial activities, such as basketball, bowling, tap dancing, navigating in a car, interior decorating, and fixing radios (also see Signorella et al., 1986; Voyer et al., 2000). However, this line of studies focused on one’s preference for spatial activities rather than competence in these spatial activities/behaviors.

Eliot and Czarnolewski (2007) designed an Everyday Spatial Behavioral Questionnaire (ESBQ) focusing on spatial competence. College students were asked to rate how often they found each activity difficult to perform. There were 116 everyday spatial activities. Factor analyses revealed 12 subscales, including object capacity, estimating covering, estimating distance, estimating direction, reversals, accurate drawing, spatial movement, driving, spatial memory, disembedding, assembling objects, and judging relationships. Canonical correlation analyses with age, sex, and different spatial tests (e.g., hidden figures) revealed two latent characteristic roots: moving through space (e.g., driving, walking) and 3-dimensional visualization. Furthermore, some spatial tests (i.e., Hidden Figures, Maze tracing test) loaded on the same characteristic root as some subscales of ESBQ. Although, some other spatial tests (i.e., Gestalt completion, Card rotation) did not.

Lawton et al. (2015) later revised the ESBQ by adding more items about movement in space (grasping vs. distance action space) and removing other items, resulting in a total of 132 items. They found 12 subscales with slightly different namings: relating objects to earth-fixed axes, movement in proximal space, navigation/orientation, fitting, driving, disembedding/targeting in proximal space, spatial relations in pictures, horizontality/verticality in proximal space, overlaying/covering space, distance/area relations, moving objects in proximal space, and following dance instructions/drawing in proportion. They also found sex differences. Women perceived some activities (e.g., relating objects to earth-fixed axes, movement in proximal space, driving, and navigation) to be more difficult than men did, but they perceived some other activities (e.g., overlaying/covering space, fitting, following dance instructions, and drawing in proportion) to be less difficult. This series of ESBQ questionnaires is instrumental in measuring competency in a variety of everyday spatial behaviors. However, these studies focused exclusively on adults. It is still unknown how competent children are at everyday spatial behaviors, how competence in everyday spatial behaviors goes through development during childhood, and their associated individual differences.

### The importance of spatial abilities and behaviors for children

Almost 70 years of research has solidified that spatial abilities are critical for developing expertise in STEM fields (Super and Bachrach, 1957; Wai et al., 2009). Project Talent, a longitudinal study tracking adolescence into adulthood, found that the likelihood of obtaining advanced STEM degrees increases as a function of spatial ability during adolescence (Wai et al., 2009). Many STEM fields depend greatly on spatial thinking and reasoning. For instance, geology may require students to mentally transform rock layers to understand how the mountain takes the shape they do (Uttal and Cohen, 2012). The field of biology may require students to understand the spatial structures of protein molecules. For many abstract scientific phenomena and concepts, students also need to comprehend and describe graphs, diagrams, and physical models which reflect visual–spatial representations. Therefore, competence in spatial thinking and reasoning may help to increase STEM success and the number of people going into STEM fields (Uttal and Cohen, 2012; Stieff and Uttal, 2015). However, unlike mathematics and verbal abilities, which are also important predictors of STEM success and formally taught at school, spatial abilities have received much less attention in the K-12 school curriculum (Kell and Lubinski, 2013).

Studying everyday spatial behaviors may open a window for us to better identify opportunities to engage and promote spatial abilities in children’s daily lives. Many studies have supported this proposition. For instance, Schug et al. (2022) found that playing with Legos in childhood predicted better performance on mental rotation tasks in adults. Similarly, Jirout and Newcombe (2015) found that parent reports of children playing with puzzles, blocks, and board games positively predicted children’s performance on the Block Design test on the WPPSI-IV (Wechsler Preschool & Primary Scale of Intelligence) for children whose parents reported they played often. Some experimental studies focusing on spatial language also lent strong support for the causal relationship between spatial experience and spatial cognition. For instance, Casasola et al. (2020) found that engaging in spatial language during play could improve children’s mental rotation performance relative to the control condition with little spatial language. A series of naturalistic, museum studies also corroborate that conversations and constructive plays, typically elicited by parents and learned by children, could help improve children’s performance on a variety of spatial tasks (Haden et al., 2014; Polinsky et al., 2017; Pagano et al., 2019). Considering the important role of spatial behaviors in spatial development, it is therefore critical to examine daily spatial behaviors in children.

### Current study

Increased competence and interest in everyday spatial behaviors may ultimately engage, motivate, and promote spatial abilities as well as STEM learning and readiness (Katz, 2011; Leyva et al., 2021). For instance, the common spatial behaviors of putting together puzzle pieces or assembling furniture encourage spatial thinking and reasoning. These types of spatial thinking and reasoning are relevant to many STEM problems, such as understanding the structures of DNA and atoms. However, few studies have examined everyday spatial behaviors in children, especially young children who have just started formal learning of spatial concepts such as maps. Several studies have probed a series of informal STEM-related activities (Ramani et al., 2015; Zucker et al., 2021; Hightower et al., 2022) and included certain spatial-related items (e.g., talking about shapes, playing with blocks), but did not focus on spatial behaviors exclusively. Many studies that had examined everyday spatial behaviors comprehensively only examined college students (Eliot and Czarnolewski, 2007; Lawton et al., 2015). A few have examined childhood spatial activities but have used retrospective reports from adults, which is prone to recall bias (Lawton and Kallai, 2002; Vieites et al., 2020; Schug et al., 2022). Therefore, the current study aimed to fill this gap and investigate parent reports of everyday spatial behaviors in children aged 4–9 years old in order to examine informal STEM-related activities during early childhood. Parent reports such as the one used here have been increasingly used to examine cognitive development. For instance, Yang et al. (2018) found parents reported that their children with intellectual disabilities (i.e., Down Syndrome) with a mental age of 4–9 years old have few wayfinding skills but much confidence. Hence, children’s limited metacognitive abilities (Salles et al., 2016) can make parent reports a highly useful tool to investigate everyday spatial behaviors. Furthermore, parent reports take less time and resources than observation-based studies and can examine multiple and diverse spatial behaviors of children in one setting.

Studying everyday spatial behaviors may help demonstrate to parents and other stakeholders that many real life behaviors involve spatial skills and these everyday spatial behaviors may be a fertile ground for spatial concepts and skills to germinate in children. In fact, many parents may not realize that children can develop cognitive skills and learn science during play and daily activities (Gomes and Fleer, 2019). Studying everyday spatial behaviors in children may also contribute to understanding individual differences in spatial abilities. Spatial ability is one domain where researchers have found relatively strong evidence of sex differences indicating a male strength (Johnson and Meade, 1987). However, the origin, cause, and development of these sex differences are still under debate (e.g., Levine et al., 2005; Newcombe, 2020; Rahe and Jansen, 2022). Vieites et al. (2020) found that childhood wayfinding experience (e.g., distance traveled) could mediate sex differences in some wayfinding strategies (i.e., route, but not survey) and anxiety in adults (also see Lawton and Kallai, 2002; Schug et al., 2022). Therefore, studying spatial behaviors in children may help understand whether vast individual differences observed for many spatial abilities also extend to everyday spatial behaviors. This knowledge may help identify the behavioral precursors of individual differences in spatial abilities in adults and inform training programs to improve spatial abilities and STEM-related competence (Stieff and Uttal, 2015).

In the current study, parents were asked whether their children were competent in a series of everyday spatial behaviors. To situate everyday spatial behaviors in a broader developmental context, we also examined the relationship between everyday spatial behaviors and other childhood outcomes, including adaptive behaviors, cognitive ability, and sense of direction. Lastly, we examined age- and sex-related individual differences to determine whether increasing age was associated with increasing competence in everyday spatial behaviors and whether boys and girls differed.

## Methods

### Participants

This study was part of a larger study that examined spatial abilities and behaviors in developmental populations. A total of 174 children aged 4–9 years old (97 boys and 77 girls) completed a series of cognitive and behavioral tests. All but one was free of any intellectual or developmental disabilities as reported by their parents (see results section). One parent identified their child as having Autism. Their data are included because removing or keeping their data did not impact the pattern of results. For more detailed information about age and sex composition, please see Table 1. One parent of each child participant completed the questionnaires. There were two testing modalities: in person vs. online. Earlier participants (65 children and 65 parents) completed the study in person. Due to Covid, later participants (109 children and 109 parents) completed the study online. Differences between the two modalities are discussed in the results section. Participants were recruited through listservs, local programs (e.g., afterschool programs, street fairs), social media (e.g., Facebook, Instagram, *ChildrenHelpingScience.com*), and lists of previous participants. In-person participants received a $40 Amazon gift card. Online participants received a $50 Amazon gift card because of longer sessions. All the recruitment and testing procedures followed the ethical guidelines of the university.

**Table 1:** Sex and age distribution of child participants.

Age group	*N*	# of Males	# of Females	Mean (age)	SD (age)
4.00–4.99	32	15	17	4.51	0.30
5.00–5.99	41	27	14	5.49	0.28
6.00–6.99	23	13	10	6.49	0.32
7.00–7.99	23	13	10	7.50	0.29
8.00–8.99	23	12	11	8.51	0.29
9.00–9.99	32	17	15	9.55	0.27

### General measures and procedures

Parents completed a demographic questionnaire (about their children’s age, sex, and presence/absence of intellectual/developmental disabilities), Everyday Spatial Behavior Questionnaire for Children (ESBQC), Santa Barbara Sense of Direction Scale (SBSOD), and Vineland Adaptive Behavior Scales (Vineland-3), in that order. All the questionnaires were presented online *via* Qualtrics. For in-person testing, parents completed the questionnaires independently on a computer in a quiet lab room, while their children completed the testing in a separate room with one or two researchers. For online testing, parents completed the questionnaires on their own time before their children started the testing with the researchers over zoom. Children completed a series of cognitive and behavioral measures. They always completed the Raven’s Progressive Matrices test (Raven’s 2) first. Only Raven’s 2 from the child measures was used here.

#### Parent measures

##### ESBQC

To build the ESBQC, we first obtained the 36 items from ESBQ published in Lawton et al. (2015). We removed items inappropriate for children, such as those related to driving and parking. Three trained research assistants worked independently and collaboratively to generate 31 new items. These newly generated items were similar to the original items and also complemented the existing ones. For instance, in Lawton et al. (2015), one item is “folding laundry” and we generated a similar item of “making a bed (i.e., evenly spreading sheets over the mattress).” Based on two items about ball-related sports (e.g., “Hitting an easily tossed ball with a bat or racket,” Lawton et al., 2015), we generated several similar items such as “Catching a ball someone has thrown at them.” One item in Lawton et al. (2015) is “Retracing a route backwards through an unfamiliar city.” Based on this item, we expanded it into three items related to navigation:

1. *Retracting a route backwards through an unfamiliar place (e.g., the parked car on an unfamiliar playground)*
2. *Retracting a route backwards through an unfamiliar place (e.g., the entrance of an unfamiliar mall)*
3. *Retracting a route backwards through a familiar place (e.g., retracing their steps back to the front door in a familiar store or house)*

Among these three items, the first is about navigation in unfamiliar outdoor environments, the second is about navigation in unfamiliar indoor environments, and the third is navigation in familiar environments. Previous research has found that spatial navigation is different in different environments for children (Yang et al., 2022).

Extensive discussions were carried out to ensure that these items (1) were appropriate for our participants, (2) reflected common, everyday spatial behaviors, and (3) complemented the existing items. The reading level of all items was chosen to be at an 8th-grade level, confirmed through readability tests. One parent of a 5-year-old was invited to pilot test ESBQC for reading level, ambiguity, and appropriateness. The ESBQC final version consists of 52 items. Among them, 46 items are appropriate for children between 4 and 9 years old. There were 6 unique items for adolescents and adults with intellectual and developmental disabilities, which are not used in this study.

Parents were given the following instructions, slightly modified from the original ESBQ adult version (Eliot and Czarnolewski, 2007; Lawton et al., 2015).

Please rate the perceived difficulty of the behaviors listed below based on your child’s prior experience with the behaviors or similar behaviors. If your child has not engaged in one of the behaviors listed, imagine how difficult your child would find the activity based on their ability with other, similar activities. Please indicate whether your child always, very often, sometimes, rarely, or never has difficulties with these behaviors by clicking the button that corresponds to each answer.

Their rating was on a 5-point Likert Scale (1: always difficult; 5: never difficult). See Table 2 for all the items.

**Table 2:** Pattern matrix based on the principal component analysis.

	Component							
	1	2	3	4	5	6	7	8
Touching a smudge on their face while looking in the mirror	0.724							
Judging whether a hole is vertical	0.620							
Being able to tell which of two objects in the room is closer to them	0.542							
Ability to judge if water will spill out of a glass when tilted	0.541							
Deciding whether they have drawn a perfectly horizontal line on a blank piece of paper	0.514							
Moving their left or right hand when told to do so	0.510							
Rotating an object that they are carrying (i.e., a large box or wide toy) so that it can fit through a smaller door	0.465							
Estimating how far apart two outdoor places are from each other (i.e., how far is the store from the car or the playground from the school)	0.451							
Deciding whether a cut out shape will fit into a hold (i.e., is the shape the right size to fit the hole)	0.434							
Judging whether a picture is straight when hung on a wall	0.418							
Kicking a ball that was kicked toward them (e.g., kicking a soccer ball)		0.851						
Hitting an easily tossed ball with a bat or racket		0.829						
Catching a ball someone has thrown at them		0.818						
Identifying where a ball will land if it has been dropped from a ladder		0.530						
Correctly running toward a spot where they anticipate a ball will land after it has been thrown from a distance		0.478						
Swatting a fly		0.456						
Following a dance step as in square dancing			−0.631					
Follow dance moves from someone who is facing them (i.e., moving their right arm for a dance move even though it looks like the instructor is moving their left hand since they are facing them)			−0.606					
Pointing to the right-hand side of the person facing them			−0.500					
Walking through a doorway without knocking against it			0.451					
Finding a pen on a crowded surface (e.g., desk or table)				0.835				
Finding one object among many (e.g., a Lego among blocks or a coin among leaves)				0.792				
Helping another person find their glasses or keys				0.757				
Ability to pick out pennies from a pile of other change				0.563				
Making a bed (i.e., evenly spreading sheets over the mattress)					0.594			
loading the dishwasher (or placing dishes in a drying rack)					0.558			
Retracting a route backwards through an unfamiliar place (e.g., the parked car on an unfamiliar playground)						−0.882		
Retracting a route backwards through an unfamiliar place (e.g., the entrance of an unfamiliar mall)						−0.868		
Retracting a route backwards through a familiar place (i.e., retracing their steps back to the front door in a familiar store or house)						−0.592		0.401
Drawing objects proportionately to each other in a picture (e.g., a big house, a tree and smaller people)							0.779	
Writing inside the lines on lined notebook paper							0.754	
Drawing a person so that parts of their body are in proportion							0.656	
Drawing a 5-pointed star							0.541	
Folding a piece of paper into equal halves							0.500	
judging whether a chair is low enough to fit under a table								0.629
Deciding whether an article of clothing will fit without trying it on								0.463
Judging where North is in an unfamiliar playground								−0.410
Judging whether the corner of an object is square								
Estimating how far apart two objects are on a table								
Judging whether one thing is in front of another in a picture								
Assembling blocks or Legos to match a picture of blacks or Legos that have already been assembled								
Put puzzle pieces together								
Packing a bag or suitcase so that the bag can zip shut or putting toys in the toy box so that the lid can close								
Identifying landmarks that lead to home (i.e., a street sign or tree indicating that they are close to home)								
Using hallway signs and pictures to find their classroom at school								
Using signs or pictures to find a familiar place such as an aisle in the grocery store								
Eigenvalues (Initial)	17.453	3.026	2.402	1.670	1.622	1.402	1.122	1.064
% of variances (after rotation)	10.943	6.731	2.774	8.899	3.838	9.120	9.878	3.947
Reliability (alpha)	0.9	0.86	0.72	0.81	0.71	0.82	0.9	0.58

Factor loadings were ordered from the largest to the smallest for each factor. For ease of reading, only absolute values of coefficients over 0.40 are displayed.

##### SBSOD

SBSOD (Hegarty et al., 2002) is a self-report measure of environmental ability or sense of direction. The scale contains 15 items, has been standardized and has good validity and reliability. We modified the wording, and parents rated their children’s sense of direction on a 7-point Likert scale (1: Strongly agree; 7: Strongly disagree). For instance, we changed the original item of “I am very good at giving directions” to the modified item of “They are very good at giving directions.” We reverse-coded items when needed so that higher scores indicated a better sense of direction. Total scores were obtained as outcome measures.

##### Vineland-3

Vineland-3 Comprehensive Parent/Caregiver form (Sparrow et al., 2016) measures adaptive behaviors based on 4 domains: Communication, Daily Living Skills, Socialization, and Motor Skills. This measure has been normed and standardized, has good reliability and validity, and is suitable for participants ages birth-90 years old. Parents were asked to rate their child’s ability to perform each behavior without help. The Vineland Adaptive Behavior Composite (ABC) scores were obtained as outcome measures.

#### Child measures

Raven’s 2 (Raven, 2018) is a nonverbal test that measures general cognitive abilities. The test has been normed and standardized, has good reliability and validity, and is suitable for participants ages 4–90 years old. In each trial, child participants needed to detect a pattern among several figures and choose the correct answer. The total raw scores were obtained as outcome measures.

## Results

One parent scored the same responses (i.e., 5) for over 95% of all the questions in ESBQC. However, removing their data did not significantly alter the pattern of the results. Therefore, results were reported based on the entire sample. Some parents or their children did not finish all the questionnaires or Raven’s 2. Their data were included whenever possible. A total of 162 parents and their children have completed all measures.

### Descriptive analysis

We first conducted a descriptive analysis of the 46 items on ESBQC. The mean of each item ranged from 1.91 to 4.45. SD ranged from 1.32 to 0.69. The skewnesses of all items were all between +/−1, except for two items (picking out pennies from a pile of other change, −1.1, and telling which of two objects in the room is closer, −1.2; i.e., both left-skewed). The kurtoses of all items were all between +/−1, except for one item (telling which of two objects in the room is closer, 1.24, i.e., leptokurtic).

Among the 46 items, the 3 lowest rated (i.e., most difficult) items were:

Judging where North is in an unfamiliar playground (*M* = 1.91)
Retracing a route backwards through an unfamiliar place (e.g., the entrance of an unfamiliar mall; *M* = 2.96)
Swatting a fly (*M* = 3.16)

The 3 highest rated items (i.e., least difficult) were:

Judging whether the corner of an object is square (*M* = 4.3)
Walking through a doorway without knocking against it (*M* = 4.44)
Being able to tell which of two objects in the room is closer to them (*M* = 4.45)

### Exploratory factor analysis of ESBQC

A principal component analysis was conducted on the 46 total items with oblique rotation (Oblimin with Kaiser Normalization) in SPSS 26. The Kaiser-Meyer-Olkin measure verified the sampling adequacy for the analysis, KMO = 0.912 (Field, 2013). All KMO values for individual items were >0.78, which is well above the acceptable limit of 0.5. Bartlet’s test of sphericity *χ*^2^ (1035) = 5185.19, *p* < 0.001, indicating that the correlations between items were sufficiently large for the analysis.

The principal component analysis yielded 8 components with eigenvalues over Kaiser’s criterion of 1 and explained 64.70% of the variance in combination. See Table 1 for the pattern matrix. Only coefficients over 0.40 were displayed. We also performed reliability for each factor and the results are also listed in Table 2. We interpreted each component based on previous theoretical frameworks on categorizing spatial abilities (Lohman et al., 1987; Carroll, 1993; Montello, 1993; Newcombe and Shipley, 2015). The first component (10 items) was mainly about spatial perception in proximal space. The second component (6 items) was mainly about sports-related activities. The third component (4 items) was mainly about bodily spatial awareness. The fourth component (4 items) was mainly about spatial visual search. The fifth component (2 items) was mainly about fitting (e.g., making a bed). The sixth component (3 items) was mainly navigation. The seventh component (5 items) was mainly about drawing in proportion. The eighth component (4 items) was a mix of navigation, fitting, and spatial perception. All components except the eighth demonstrate acceptable reliability, >0.70. There were 9 items that did not have factor loadings over 0.40 on any of the component. One item (retracting a route backwards through a familiar place) loaded on components 6 and 8. We removed all the items that did not load on any components and all the items on component 8 due to low reliability. This resulted in a total of 34 items. We obtained the average score of these 34 items and analyzed its relations to other variables we collected.

### Relationships with age, sex, SBSOD, and vineland

**Figure 1:**
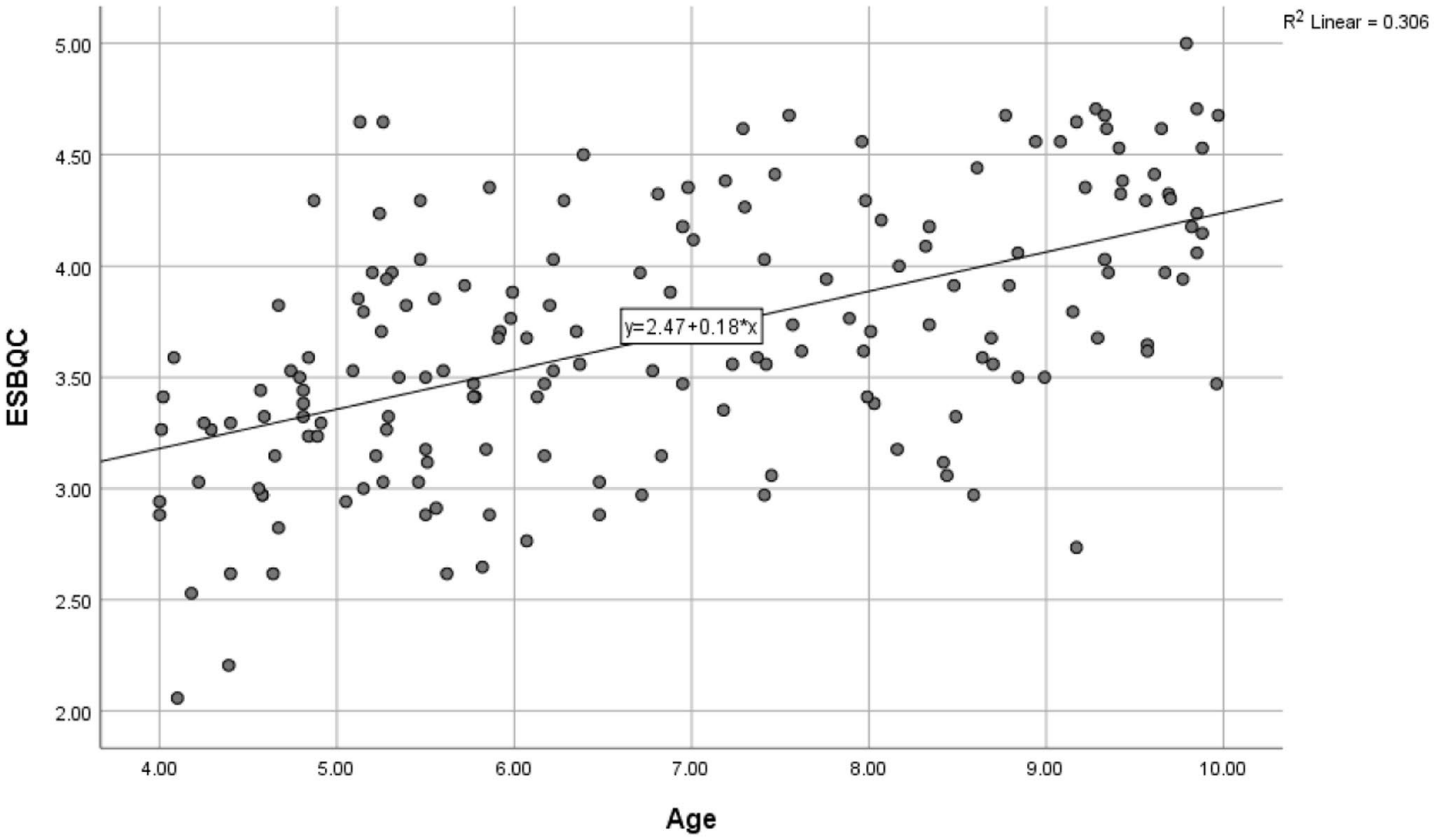
ESBQC as a function of age.

Next, we explored the relationship between ESBQC and modality, age, sex, SBSOD, Raven’s and Vineland. See Table 3. ESBQC significantly correlated with modality, age, SBSOD, Raven’s, and Vineland. The correlation strengths were moderate to large for all the significant correlations except for the one with modality (i.e., small). SBSOD was not correlated with sex, suggesting no sex differences between boys and girls in their parents’ reports of their everyday spatial behaviors. Modality was significant such that ratings were higher in person (*M* = 3.83, SD = 0.59) than online (*M* = 3.59, SD = 0.59). See Figure 1 for a scatterplot of ESBQC as a function of age. It showed that competency in everyday spatial behaviors develops as a function of age. There were also wide individual differences in each age group.

**Table 3:** Pearson correlations between variables (sample sizes varied from 162 to 174).

	Age	Modality	Sex	Raven’s	Vineland	SBSOD
ESBQC	0.553^**^	0.199^**^	−0.065	0.390^**^	0.469^**^	0.483^**^
Age	1	−0.089	0.032	0.676^**^	0.204^**^	0.156^*^
Modality		1	−0.042	−0.118	0.044	0.144
Sex			1	0.103	−0.190^*^	0.130
Ravens’				1	0.245^**^	0.156^*^
Vineland					1	0.365^**^

p* < 0.05. p** < 0.001. Sex: 0: girls, 1: boys. Modality: 0: online; 1: in person.

Next, we conducted a hierarchical linear regression analysis to examine the validity of ESBQC. First, we examined whether ESBQC could predict SBSOD, which measures spatial ability in large-scale environments. We entered age in the first step. Sex and modality were not entered because their zero-order correlations with SBSOD were not significant. In step 2, we entered Vineland. Vineland is not designed specifically to measure spatial abilities but adaptive behaviors instead. However, it is also a questionnaire that parents completed. If parents have an overall tendency to answer similarly for all questions about their children, such as deeming their children capable of all sorts of activities, then entering Vineland in step 2 would help control this measurement bias associated with parent reports. In step 3, we entered ESBQC. If ESBQC still predicted SBSOD after Vineland was being accounted for, then it would indicate that the relation between ESBQC and SBSOD was not simply because of similar measurement methods (i.e., parent report). See Table 4 for results. Most importantly, the *R*^2^ change from step 2 to step 3 was significant. Adding ESBQC was able to explain an additional 12.7% of the variance in SBSOD. Collinearity statistics showed that none of the variables had VIFs of over 10, hence there was minimal concern for collinearity.

**Table 4:** Regression results on SBSOD.

Variables	*β*	*t*	Sig.	*R* ^2^	Δ*R*^2^	*F*
Step 1				0.021		*F*(1,163) = 3.48, *p* = 0.064
Age	0.145	1.865	0.064			
Step 2				0.138	0.117**	*F*(2,162) = 13.00, *p* < 0.001
Age	0.073	0.981	0.328			
Vineland	0.350	4.699	0.000			
Step 3				0.265	0.127**	*F*(3,161) = 19.36, *p* < 0.001
Age	−0.148	−1.831	0.069			
Vineland	0.174	2.272	0.024			
ESBQC	0.472	5.269	0.000			

**p < 0.01. Criterion (dependent) variable: SBSOD.

We examined the relationship between SBSOD and ESBQC more closely. SBSOD was on a 7-Likert Scale (Hegarty et al., 2002) and ESBQC was on a 5-point Likert Scale (Lawton et al., 2015). To make the two scales comparable, we used the following formulas to transform the data:

SBSOD_rescaled = (SBSOD-1)/(5–1)
ESBQC_rescaled = (ESBQC-1)/(7–1)

**Figure 2:**
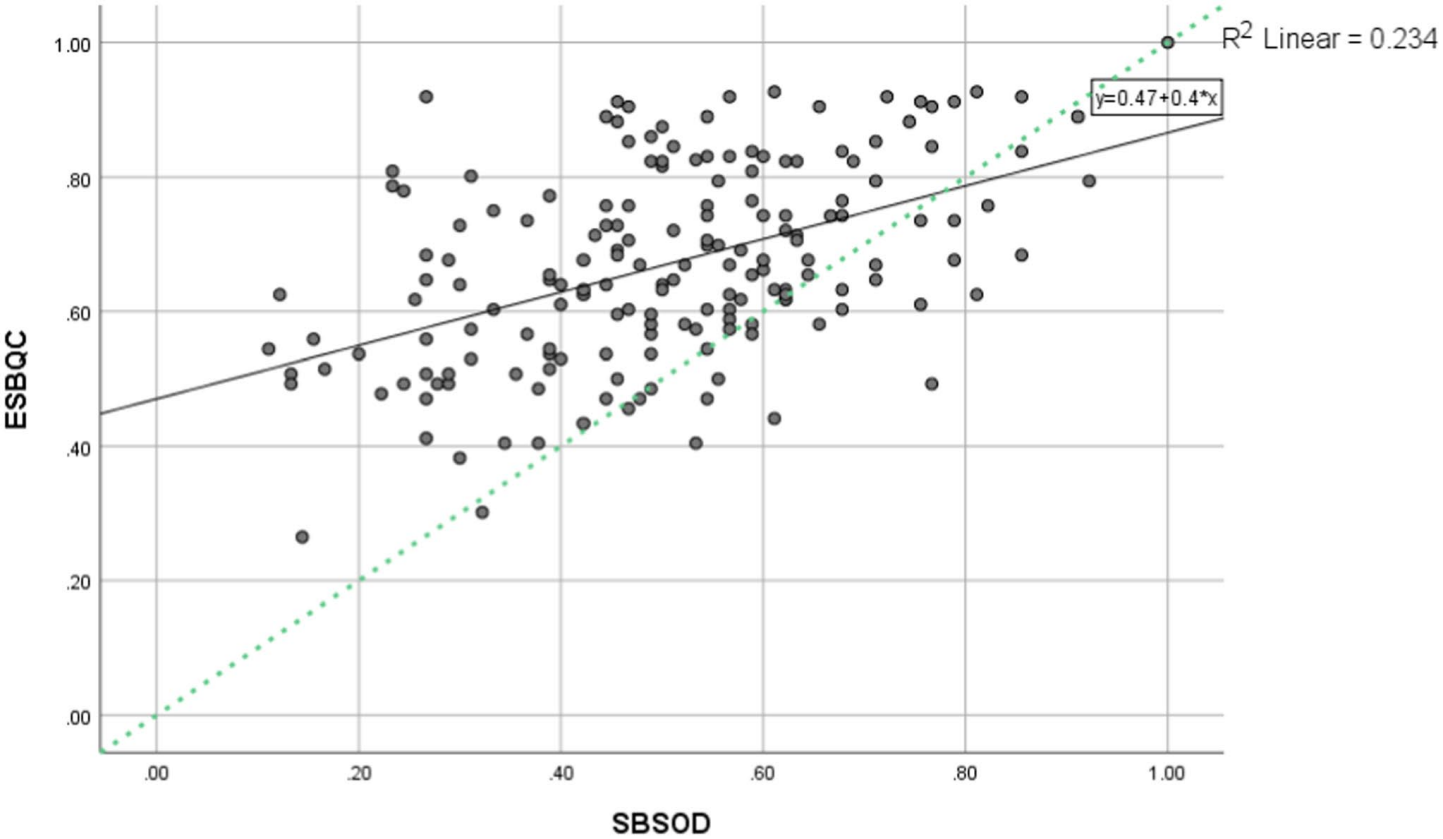
The relationship between SBSOD rescaled and ESQBC rescaled. The black solid line shows the regression line, whereas the green dotted line shows y = x.

This way, both questionnaires were on the same scale, ranging from 0 to 1, where 0.5 indicates a balance point (e.g., neither agree nor disagree). Then we plotted the two rescaled scores. See Figure 2 below. It is apparent that the relationship was positive such that increases in SBSOD were associated with increases in ESBQC. Furthermore, if the two sets of scores were perfectly aligned, they should form a perfectly diagonal line of y = x (or y = 0 + 1 * x). As shown in the figure, most scores were above the diagonal line. In other words, most children found everyday spatial behaviors on the ESBQC easier than those on SBSOD.

Finally, we examined whether ESBQC could also predict child outcomes. The zero-order correlation between ESBQC and the child measure of Raven’s 2 was significant, *r* = 0.390, *p* < 0.001. However, the partial correlation between the two after partialling out the effects of age was no longer significant, *r*_p_ = 0.046, *p* = 0.559. This also applied to SBSOD: while the zero-order correlation between SBSOD and Raven’s 2 was significant, *r* = 0.156, *p* = 0.042, the partial correlation between the two after considering age was no longer significant, *r*_p_ = 0.092, *p* = 0.248.

## Discussion

To better understand everyday activities relevant to informal STEM learning, we developed an everyday spatial behaviors questionnaire for children (ESBQC) based on prior research on adults (Eliot and Czarnolewski, 2007; Lawton et al., 2015). A total of 174 parents completed the ESBQC about their children. In addition, they completed the SBSOD and Vineland-3. Their children, aged 4–9 years old, completed the Raven’s 2, a normed measure of general cognitive ability. Exploratory factor analyses showed 8 components, accounting for over 60% of the variance. Individual differences analyses showed that increasing age was associated with higher scores in ESBQC, yet there were no sex differences in ESBQC. Correlation analyses showed that ESBQC was significantly correlated with children’s adaptive living skills, sense of direction, and cognitive ability. Regression analyses showed that ESBQC predicted SBSOD even after considering the effects of age and measurement bias associated with parent reports. However, ESBQC did not predict children’s Raven’s 2 after considering age.

### Evaluating ESBQC

Our study showed the factor structures of ESBQC, its high internal reliability, and high converging validity. Our factor analysis generated 8 components, unlike the 12 subscales in the original studies of ESBQ for adults (Eliot and Czarnolewski, 2007; Lawton et al., 2015). One difference between our study and earlier studies is that ESBQC had much fewer items (46 total) relative to the original ESBQ adult version (i.e., 116 and 132). Moreover, while ESBQC focused on parents of children between 4 and 9 years old, the original ESBQ adult version studied college students. It is also important to note the multifaceted nature of everyday spatial behaviors. One spatial behavior may involve more than one type of spatial ability. For instance, when trying to find a missing puzzle piece, one would first decide and locate where the missing piece should go, recognize the unique spatial features of surrounding pieces, store this information in visual–spatial short-memory, visually search all the loose pieces, mentally or manually rotate a certain piece to see if it fits, and repeats this process until finding one that fits. During this process, spatial perception, spatial memory, and mental rotation would all have been involved. Therefore, the factor structures of everyday spatial behaviors may be more intertwined and complicated than those from laboratory spatial tasks. Reliability analysis showed acceptable to high reliabilities for 7 out of the 8 factors. We recommend using the 34 items, excluding component 8 with low reliability and all the items that did not load on any factors. The reliability of the 34-item ESBQC as a whole was very high: alpha = 0.95.

Our study also showed reasonable converging validity with SBSOD, which itself has been validated with experimental tasks (Hegarty et al., 2002). It is also interesting to consider the differences between the two measures. Scatterplots showed that everyday spatial behaviors on ESBQC were perceived to be less difficult for children relative to navigation-specific behaviors on SBSOD. Spatial navigation as assessed by SBSOD might represent the most difficult form of spatial cognition in our daily lives because navigation requires numerous cognitive abilities such as planning, reasoning, and decision-making (e.g., Brunyé et al., 2018; Dalton et al., 2019). Due to safety concerns, children also have fewer opportunities and experiences for independent spatial navigation compared with other types of spatial behaviors (e.g., make a bed) as investigated by ESBQC.

Although ESBQC correlated with Raven’s 2 at the zero-order level, the partial correlation between the two after considering age was no longer significant. There are several reasons for the lack of significant relations after partial correlations. First, parent reports might not be reliable measures of child performance in laboratory settings. Parents may know how their children behave in daily life. However, this may not translate to a laboratory task that children have never experienced before. Second, Raven’s 2 is a general measure of cognitive ability. Other cognitive measures that directly tap into spatial abilities, such as spatial perception tasks, sports-related movement tasks, and navigation tasks, may have a stronger relationship with ESBQC. However, we think that even though neither SBSOD nor ESBQC correlated with Raven’s 2 after partialling out age, it does not necessarily diminish the utility of ESBQC. ESBQC is simply not measuring the same psychological construct that Raven’s 2 is measuring. In fact, many daily behaviors, habits, and activities are better off being measured by questionnaires with higher ecological validity than cognitive tasks with high internal validity but much limited ecological validity. Future research should continue to explore the predictive validity of ESBQC.

### Everyday spatial behaviors and informal STEM learning

We hope that ESBQC may help show parents and other stakeholders the wide array of everyday spatial behaviors that children are engaging in or could engage regularly. Often, in the eyes of a layperson, there is a disconnect between laboratory studies of spatial abilities and people’s everyday experiences. Previous research has found that many parents may not recognize daily opportunities to engage their children in informal STEM learning (Gomes and Fleer, 2019; Zucker et al., 2021). For instance, mental rotation is widely understood by spatial cognition researchers but seems jargony, or at least unrelatable, to people outside the academe. Some common mental rotation examples in adult life could involve installing ink toners or assembling furniture. However, there is very little chance that 4-9-year-old children would engage in these behaviors. Our ESBQC included specific real-life examples that children may engage such as “Deciding whether a cutout shape will fit into a hold” and “Rotating an object that they are carrying (e.g., a toy) so that it can fit through a smaller door.” ESBQC may be used in broader contexts outside of research. For instance, teachers and other educators can use examples from ESBQC to show parents ample daily opportunities to encourage children’s spatial behaviors at home. ESBQC may also be used to identify different students’ strengths and weaknesses, which would then help advise ways for more individualistic educational plans for STEM learning.

Identifying spatial behaviors in everyday settings in the first place may lead to training and teaching moments to promote spatial abilities and activities (Ramani et al., 2015; Pagano et al., 2019). This is particularly relevant to early childhood when children are not equipped to grapple with more complex spatial thinking and reasoning concepts in formal curricula. For instance, when playing with Legos or puzzles, parents may help children recognize 2D and 3D shapes and engage in spatial rotation, transformation, and imagery. When navigating in an unfamiliar environment, parents may help children notice the geometric structures of buildings and study the layout of the streets. The interests, engagement, and motivation developed early in childhood may also help develop the formation of STEM identity and learning and readiness later on (Maltese et al., 2014; Dou et al., 2019). For instance, the everyday spatial behavior of exploring and navigating in an unfamiliar spatial environment may encourage the development of wayfinding skills and interests relevant to STEM careers such as airplane pilots and architects. It may also promote understanding basic principles of urban design and geography. Overall, our results indicate the availability of ample opportunities to engage in informal STEM learning (Katz, 2011; Leyva et al., 2021) through everyday spatial behaviors, the potential of which might have yet to be fully explored by parents, educators, and other stakeholders.

### Individual differences in everyday spatial behaviors

We found an age effect such that an increase in age was associated with better everyday spatial behaviors. The correlation coefficient between age and ESBQC was numerically the largest compared with correlation coefficients that ESBQC had with other variables. The age effect was expected and consistent with previous laboratory studies of spatial cognition in children (e.g., Merrill et al., 2016; Nazareth et al., 2018). There might be a bi-directional relation between spatial competency and spatial behaviors/activities frequency. Children’s increasing competency in everyday spatial behaviors may encourage them to engage in more spatial behaviors in more contexts, which in turn further facilitates the improvement of their competency in everyday spatial behaviors. Social–emotional factors such as confidence may also play a role such that increasing competency in everyday spatial behaviors makes children more confident in their abilities, and this increased confidence leads to more activities and better competency. For instance, a young child who is good at Legos may continue to play more Legos as they grow older and become more confident in their spatial construction skills, leading to an even higher competency in Legos playing. These increased experiences, confidence, and competency in everyday spatial behaviors may facilitate the development of spatial cognition (Vasilyeva and Lourenco, 2012; Jirout and Newcombe, 2015; Schug et al., 2022) and STEM success (Uttal and Cohen, 2012; Stieff and Uttal, 2015).

Despite previous research finding sex differences in everyday spatial behaviors in adults (e.g., Newcombe et al., 1983; Lawton et al., 2015), we did not find a sex difference in children aged 4–9 years old. A meta-analysis has found that effect sizes of sex differences in spatial wayfinding were typically smaller for children younger than 13 years old than for adults (Nazareth et al., 2019). Admittedly, boys often demonstrate strength compared with girls in certain laboratory tasks measuring wayfinding and mental rotation (Hoyek et al., 2011; Jansen et al., 2013; Merrill et al., 2016). However, in real-life situations, girls may have a repertoire of strategies available to compensate for a possible shortcoming in spatial abilities, if the shortcoming does exist. For instance, putting together puzzle pieces can benefit from attention to detail and visual processing (Powers et al., 2013) in addition to the assumed underlying spatial abilities such as spatial perception and mental rotation skills. Furthermore, in real-life situations, experience and opportunities to engage in spatial behaviors may play a bigger role than the assumed cognitive abilities. For instance, although a new student may need help finding their classroom on the first day of a new school, they typically have no trouble after one semester.

We also found that modality had a small correlation with ESBQC, such that in-person parents rated their children more favorably than online parents. This result was unexpected. It is possible that in-person parents have a stronger motivation for social desirability in front of the experimenters than online parents (however, see Dodou and de Winter, 2014). There are also alternative possibilities, such as the impact of Covid on parental stress (Adams et al., 2021). Future research can explore the online vs. in-person difference in more detail.

## Conclusion

Spatial thinking is critical to STEM success because many STEM problems involve spatial thinking and reasoning (Uttal and Cohen, 2012; Stieff and Uttal, 2015). Spatial cognition is also highly malleable (Uttal et al., 2013; Stieff and Uttal, 2015; Reilly et al., 2017) and can be improved through experience, practice, and instructions. However, it is not typically taught in the K-12 curriculum (Kell and Lubinski, 2013). By examining everyday spatial behaviors in children aged 4–9 years old, our study showed that there are many daily opportunities to engage, motivate and promote informal STEM-related activities during early childhood. We hope our study will encourage more attention, interest, and awareness of informal STEM-related activities in future research.

## Data availability statement

Data are available on request to the corresponding author.

## Ethics statement

The studies involving human participants were reviewed and approved by Institutional Review Board, Montclair State University. Written informed consent to participate in this study was provided by the participants’ legal guardian/next of kin.

## Author contributions

YY contributed to the conception, design, and analysis of the study and wrote the first draft of the manuscript. SC organized the database, analyzed the data, and contributed to sections of the manuscript. DL, AH, and LG coordinated the study, collected data, and organized the database. LG contributed to the design of the study. All authors contributed to the article and approved the submitted version.

## Funding

This project was in part supported by NIH Grant # SC2HD103587 awarded to YY.

## Conflict of interest

The authors declare that the research was conducted in the absence of any commercial or financial relationships that could be construed as a potential conflict of interest.

## Publisher’s note

All claims expressed in this article are solely those of the authors and do not necessarily represent those of their affiliated organizations, or those of the publisher, the editors and the reviewers. Any product that may be evaluated in this article, or claim that may be made by its manufacturer, is not guaranteed or endorsed by the publisher.
